# The harm principle, personal identity and identity-relative paternalism

**DOI:** 10.1136/jme-2022-108418

**Published:** 2023-01-20

**Authors:** Dominic Wilkinson

**Affiliations:** 1 Oxford Uehiro Centre for Practical Ethics, University of Oxford, Oxford, UK; 2 Newborn Care, John Radcliffe Hospital, Oxford, UK

**Keywords:** paternalism, ethics- medical, philosophy- medical

## Abstract

Is it ethical for doctors or courts to prevent patients from making choices that will cause significant harm to themselves in the future? According to an important liberal principle the only justification for infringing the liberty of an individual is to prevent harm to others; harm to the self does not suffice.

In this paper, I explore Derek Parfit’s arguments that blur the sharp line between harm to self and others. I analyse cases of treatment refusal by capacitous patients and describe different forms of paternalism arising from a reductionist view of personal identity. I outline an Identity Relative Paternalistic Intervention Principle for determining when we should disallow refusal of treatment where the harm will be accrued by a future self, and consider objections including vagueness and non-identity.

Identity relative paternalism does not always justify intervention to prevent harm to future selves. However, there is a stronger ethical case for doing so than is often recognised.

## Harmful choices

When we know that someone is making a choice that will predictably risk or cause him to suffer significant harm, we have a basic duty of beneficence to try to prevent that.^1^ If James (box 1) was our friend or family member, we should try to discourage his choice and promote a better alternative. If we have a professional relationship with the person, (eg, if we are James’s doctor), we would have an additional professional responsibility to advise against this decision.^1^

Box 1 **Case: vaccination refusal**

James is in his late 50s and has a number of health problems. He would be at risk of becoming seriously unwell if he were to contract COVID-19. Earlier in the pandemic, James had a number of friends and family members become seriously ill and two died. However, when James is offered a COVID-19 vaccine, he refuses. He has come to believe that the vaccine contains a microchip that would allow him to be tracked. Despite all evidence to the contrary, James persists in his belief and resolutely refuses to be vaccinated.

But what if the person persists in his choice against our advice? Should we take further steps to restrict him? Should we restrain him or forbid him from making this choice? Should James’s employer insist on him being vaccinated to continue to work? Would a state be justified in mandating vaccination?

In terms of the law, and intervention by the state, one oft-cited response draws on an important liberal principle articulated by the philosopher John Stuart Mill. Mill’s ‘harm principle’ claims that the only justification for infringing the liberties of an individual is to prevent harm to others; harm to the self does not suffice. (Mill, p23)^2^ For debates about vaccination mandates in the COVID-19 pandemic, this means that ethical arguments have focused entirely on the effect of vaccination on risk of transmission to others, or on the use of scarce hospital resources. However, as the vaccination rate in the wider population has increased, and the pressure on hospitals has abated, we might return to the paternalistic reason for wanting James to be vaccinated.^a^ Setting aside any question of harm to others, it would be far better for his own health for James to receive the vaccine.^3^ Could that possibly justify intervention to coerce or mandate vaccination?

The harm principle provides a simple and firm bolster against paternalism, against others’ well-meaning interference in our own lives. But it draws a sharp line between decisions that are harmful to the self and those that are harmful to others. It seems to imply that these are radically different types of decision and demand different ethical responses.

In this paper, I explore some reasons suggested by the philosopher Derek Parfit for dissolving or blurring the distinction between harm to self and harm to others.^4^ On Parfit’s account, some apparently self-harming decisions are relevantly like harming someone else.^b^ He noted that his reductionist account of personal identity might be considerably more permissive of paternalism than traditional ethical approaches, though did not clearly identify if paternalism would be justified in cases like that of James. In the paper, I identify two different versions of reductionist paternalism, according to which the harm principle is undermined and health professionals and states may be justified in being paternalistic in a wider range of cases. The reductionist might claim that paternalism is more easily justified, or alternatively that what is conventionally thought of as hard paternalism is not actually ‘paternalistic’. Although I will take the first approach in this paper, the second would be equally plausible.

I also explore the relevance of Parfit’s ‘wide value-based objective view’ of reasons and suggest that this supports what I call Identity Relative Paternalism. To my knowledge, this paper is the first to draw a connection between Parfit’s later writing on the nature of reasons (his wide-value-based objective view), and his earlier work on personal identity. I will focus on hard paternalism, since it is here that the reductionist account of identity may have most radical implications,^c^ and will not discuss specifically other arguments in favour of paternalism.^d^

I will focus here on medical examples and refusal of treatment by patients, identifying a spectrum of cases where relations of psychological connectedness and continuity might hold to stronger or weaker degrees despite intact cognition and in the absence of brain damage. One reason for focusing on patient refusals of treatment is that these typically (vaccination is an exception) are interpreted as being associated with harm only to the individual and not to other people. In cases where patients demand treatment (that the doctor believes will cause harm to the patient and is tempted to paternalistically refuse to provide) there may often be implications for resources that could potentially have impacts on other patients and provide a separate justification for refusal.^e^

Much has been written previously on cases involving significant changes in personality and cognition—particularly in relation to the validity of advance directives in dementia.^5–9^ For this paper, I will briefly outline some possible implications of Identity Relative Paternalism for these cases in the section on practical implications, but will not discuss dementia cases in detail. That is partly because I am interested in exploring the wider potential implications of a reductionist account of identity for paternalism (ie, beyond cases of brain injury or dementia). Second, questions about advance decisions and dementia are complicated by potential changes in moral status and lack of capacity in the later time points, so the individual’s contemporaneous views or interests might or might not be taken to have lesser ethical weight than the prior individual’s.

## The harm principle

the only purpose for which power can be rightfully exercised over any member of a civilized community, against his will, is to prevent harm to others. His own good is not a sufficient warrant (Mill, p23)^2^

Mill articulated his harm principle early in his work, ‘On Liberty’. Mill was writing specifically about the justification for government actions and in particular the important limits on government interference in individuals’ lives. His harm principle warns against enacting paternalistic laws—ones that are designed to restrict individuals’ choices for their own good. The same principle, though, can be extended to other situations—for example to medicine. It provides a strong rebuttal against doctors who might be inclined to make paternalistic decisions motivated by concern for patients’ well-being. According to the harm principle, doctors should not limit patients’ freedoms for the sake of the patient’s own good, though they may be justified in doing so if the patient’s actions would cause harms to other people.^f^

Mill was forthright about the absolute freedom of individuals to make decisions that would affect only themselves: ‘Over himself, over his own body and mind, the individual is sovereign’. (Mill, p22)^2^ ^g^ In contrast, ‘[a]cts injurious to others require a totally different treatment’. (Mill, p140)^2^ ‘The distinction between the loss of consideration which a person may rightly incur by defect of prudence or of personal dignity, and the reprobation which is due to him for an offence against the rights of others…makes a vast difference both in our feelings and our conduct towards him’. (Mill, p141–2)^2^

Mill provided two key arguments against paternalism. The first was based on the value for individuals of freedom to have opinions and to act. Such freedom is instrumentally valuable because it enriches individual lives and allows the development of human faculties. ‘The free development of individuality is one of the leading essentials of well-being’. (Mill, p102)^2^ The second was on the basis of fallibility of paternalistic judgements. Such judgements are necessarily based on presumptions that may be mistaken. (Mill, p1150)^2^ The individual’s own self-knowledge is much more reliable.^h^ I will return to these arguments against paternalism.

## Harmful decisions

To help identify the relevance of these ethical arguments it will be useful to have some practical examples. I have mentioned already the case of James and his vaccination refusal. Box 2 and box 3 illustrate two more.

Box 2 **Case: Hospital birth refusal.**

Jenny is pregnant and expecting her first child soon. Her obstetrician expresses concern that because of the position of the baby (transverse lie) a caesarean would often be required and a home birth would be very high risk for both Jenny and her baby. However, Jenny is clear that she does not wish to have a caesarean section in any circumstances. Jenny explains that she has been part of an online forum, and she strongly wishes to have a natural vaginal home birth. She has plans to ‘free birth’ and deliver in her home (which is a long way from the hospital) without any involvement of health professionals.^34^

These three cases (boxes 1–3) involve adults making decisions in relation to their own life. For the sake of argument, I will assume that at the time of the decisions, each of these patients had capacity—that is to say that they had no disorders affecting their thinking or ability to reason. If formally assessed, it would be clear in each case that they were able to understand and retain the relevant information provided to them, weigh the reasons and communicate their choice.^10^

Box 3 **Case: advance resuscitation refusal.**

John is 50 years old and is otherwise healthy. In his 20s, John became briefly infatuated with a novel that involved a main character who made decisions based on the rolling of a dice.^27^ ^35^ At the time John completed an advance directive on the basis of dice rolls. The advance directive indicated that if in the future he were to have a cardiac arrest he would not wish to be resuscitated. John registered this advance decision with his doctor at the time, but has not discussed it since. He stopped rolling dice for decision-making a long time ago, but has never revisited his advance directive. John collapses suddenly at a party.

In these cases, many might be inclined to question the rationality of the decisions made. The risks that James, Jenny and John are taking with their own health appear considerable and the reasons that they cite would appear (to most people) to be insufficient. Health professionals might try hard to change the patients’ mind. They might even seek a formal psychiatric evaluation. Nevertheless, it is likely that the decisions would ultimately be respected.^9^ After all, that is what respect for the harm principle and for patient autonomy is thought to require.

By way of contrast, we could imagine the following cases (boxes 4–6).

Box 4 **Case: Third party vaccination refusal**

As before, James is an adult with underlying health problems. However, in this version of the case, James has a long-standing intellectual disability and is non-verbal. James’ mother and long-term carer, Mary, expresses a strong desire that James not have a vaccine on the basis of her belief that these vaccines contain microchips that would connect James to a 5G network.

Box 5 **Case: Third party hospital birth refusal**

Jenny is pregnant and expecting her first child soon. Jenny has severe agoraphobia and has not left her home in 3 years. She has been assessed to lack capacity to make decisions about place of childbirth and the case has been referred to court. The judge, Michelle, has been taking part in online forums about natural childbirth, and on that basis decides for Jenny to have a free birth.

Box 6 **Case: Third party resuscitation refusal**

John is 50 years old and is otherwise healthy. His husband, Michael has recently become infatuated with a novel about dice rolling for decisions. When John collapses suddenly at a party, Michael rolls a dice and based on the result asks others present not to call an ambulance.

In these versions of the cases it seems clear that the third party decision makers would not and should not be permitted to make these harmful choices.^10^ The mother’s refusal of a vaccine for James should be overruled. The judge’s decision should be urgently appealed. Someone else present when John collapses should call the ambulance against Michael’s wishes. It is clear in the third-party versions of these cases that even if these decision-makers are autonomous adults, their decisions will cause harm to other people, and on that basis, should be overruled.

But is there such a sharp divide between the first-person and third-person versions of these cases?^11^

## Identity and paternalism

In his book, Reasons and Persons, the Oxford philosopher Parfit famously defended what he called a ‘reductionist view’ about personal identity. (Parfit, p199–345)^4^ Parfit argued that the continued existence of an individual over time can be reduced to certain physical or psychological continuities. On this view, we can identify whether and to what extent someone at time point t1 is physically and/or psychologically continuous and connected with a person at a later time point t2. For example, we can ask ‘do they share the same body, the same memories, the same patterns of thought and character traits’? According to Parfit, the answers to these questions will tell us how the earlier person is related to the later person, and what is more will tell us everything that matters.^12^ Although we might be tempted to ask ‘but is it the same person at t1 and at t2?’, according to Parfit, this question is sometimes empty. (Parfit, p213–4)^4^ At least in some special cases, there is no separate answer to this question. Once we have identified the relevant connections there is nothing additional to meaningfully say.

Should we be reductionists about personal identity? Parfit’s argument for reductionism is complex,^12^ and based on thought experiments involving divided brains and teletransportation. It is outside the scope of this paper to fully outline these arguments or to defend them in detail. However, it may be helpful to set out a brief intuitive case in favour. When someone has a profound brain injury and permanently loses the capacity for consciousness, family members often report that the person who they were has ‘gone’.^13^ There is a strong sense in such cases that even if the individual’s body remains alive, that the loss of psychological capacities is the end of their existence. In other cases, brain disorders can lead to profound changes in behaviour and personality. For example, in 2003, a 40-year-old school teacher was found to have an egg-sized tumour in his right frontal lobe.^14^ The teacher (having never previously behaved in such a way) had developed progressive uncontrollable sexual urges including paedophilia over a period of 3 years, and was diagnosed with a brain tumour only the day before planned prison sentencing. His sexual urges abated after resection of the tumour. What should we say about such a case? It seems highly plausible that the individual who made sexual advances to his prepubescent daughter was different in an important way from his earlier (and then postresection) self. That seems highly relevant to an understanding of whether we should hold the teacher responsible for the past behaviour. In these two cases, there might be disagreement about whether the person still exists after the brain injury, whether it is the same person with or without the brain tumour. But one thing that we should agree on is that the personality loss or change matters profoundly.

The reductionist view about personal identity has a number of striking implications^13^. One is that rather than thinking of oneself as a single self, existing from birth to death, it may be useful and natural to conceive of having ‘successive selves’. (Parfit, p305,19)^4^ Literature and colloquial language sometimes refers to ‘an earlier self’ or ‘a later self’. In usual circumstances, there will not be clear boundaries between these, but they are nevertheless distinct in important ways.

A second implication is that our concern about our own future (our egoistic concern) may not be binary (all or nothing)—instead it is a matter of degree.^14^ So, the person A at time point t1 can be closer to the later person B at t2, or further away—depending on the extent of psychological connectedness. The relevant question to ask is—‘how closely is A related to B’?, rather than ‘Is A identical to B’?

There are also a number of moral implications of this view about personal identity. One is that the boundary between the self and others is less distinct and less important. (Parfit, p338)^4^ Conventionally, decisions that affect only ourselves are taken to be outside the scope of morality. (Parfit, p319)^4^ It might be unwise, or even irrational to make a decision that will cause future harm to ourself, but (on a standard liberal view) it is not a question of morality. (Feinberg, 56)^15^ Parfit rejected that. He claimed that because the future self is relevantly like a different person, we should think of decisions that affect them in the same way that we think about decisions that will affect a different person. This gives rise to the Parfitian claim about what we morally ought to do

Reductionist Identity Moral Claim: “If we now care little about ourselves in the further future, our future selves are like future generations…Like future generations, future selves have no vote, so their interests need to be specially protected… We ought not to do to our future selves what it would be wrong to do to other people”,(Parfit, p319–20)^4^

In a very short section of his book, Parfit explicitly extended this to defend paternalism. He claimed that coercion or infringement of someone’s autonomy could be justified to prevent the individual from causing great harm to himself for no good reason. While we cannot justify restricting someone’s personal freedom on the grounds that they are acting irrationally, this is justified if they are acting wrongly. Parfit claimed that individual autonomy does not outweigh such moral concerns:

‘Autonomy does not include the right to impose on oneself, for no good reason, great harm’. (Parfit, p321)^4^ He went on, restating the moral claim in terms of the obligations of other people to prevent imprudence

Reductionist Identity Paternalism Claim: “We ought to prevent anyone from doing to his future self what it would be wrong to do to other people” (Parfit, p321)^4^ ^15^

Parfit briefly acknowledged two standard objections to paternalism—that it is good for people to be able to learn from their mistakes, and that generally the individual will be in a better position than others to know what is best for him or her. These objections (which we could call the consequentialist and epistemic objections) are closely related to the arguments given by Mill and noted above. But in Reasons and Persons, Parfit did not clarify whether he thought that these objections outweighed the arguments that he set out in favour of paternalism given a reductionist account of identity. He provided no clear answer to the cases of James, Jenny and John.

## Identity and treatment refusal

One point to note about the consequentialist and epistemic objections to paternalism is that they may not always apply. For example, there is little personal learning possible from a fatal error. (That would appear to potentially apply to the treatment refusal cases outlined above.) There will also be cases where we can be confident that the individual is mistaken about their own interests. James, Jenny and John in the treatment refusal cases are making serious errors of judgement. If we respect their decisions, that is not because we think they might be correct, but rather because they have a right to act imprudently. Furthermore, when we compare the first person with the third person versions of the cases, it is clear that neither the consequentialist nor the epistemic objections would come close to justifying third party harm. No matter how strongly we support their freedom to develop and have their own opinion or their knowledge of the individuals concerned, we would not allow Mary, Michelle or Michael to harm others seriously as a consequence.

Does the Reductionist Identity Paternalism claim apply to treatment refusal? There are two possibilities. One is that this claim applies equally to all instances of self-harm. It would apply equally to decisions that individuals make that affect them in the very near future, as it does to decisions that individuals make that affect them in the distant future. We could call this

Time-neutral paternalism: Individuals should be prevented from doing to themselves (whether in the near or in the further future) what it would be wrong for them to do to others.

However, as noted, Parfit’s view was that what matters morally is a function of psychological continuity and connectedness over time. This might suggest an alternative version.

Identity relative paternalism: Individuals should be prevented from doing to future selves (where there are weakened prudential unity relations between the current and future self) what it would be wrong for them to do to others.

On this identity relative account, our response to cases may vary—depending on the relationship between the person making the decision and the later-self harmed by it.

For example, in the three cases described at the start of the paper there appear to be different degrees of connection between the individuals making decisions, and their future selves harmed by their decision. In Vaccine refusal, there is likely a short period of time between when James_t1_ makes a decision and James_t2_ potentially comes to harm because of contracting severe COVID-19. This means that there are strong psychological connections between the different Jameses. In contrast, in Advance resuscitation refusal, many years have passed since John_t1_ made his somewhat rash advance treatment decision. Although John_t2_ has psychological connections with his previous self, we might suspect that they are somewhat weak. It was an earlier self (in his transient Dice Man phase) who made the advance decision to refuse treatment. John_t2_ is likely to have many different interests, tastes, preferences and priorities from John_t1_. The things that were important to him then are likely to be much less so now. *Hospital birth refusal* lies somewhat in between. In this case, there is a short temporal distance between the pregnant Jenny_t1_ and the later Jenny_t2_ who would potentially suffer a catastrophic complication of childbirth. However, the things that the pregnant Jenny_t1_ may prioritise and value could be significantly different from those of her later self. That is because some life events can profoundly alter our perspective. Such events are sometimes described as ‘transformative’. The philosopher Laurie Ann Paul provocatively imagined becoming a vampire. (Paul, P1–4)^16^ Overnight, someone’s way of life, their viewpoint, their values and preferences would transform. Such an experience might radically undermine our ability to make informed choices (because of our difficulty in imagining what life would be like). But on the Reductionist Identity account, it might also suddenly and significantly weaken the psychological connections that are morally significant. ‘[W]hen there has been a significant change of character, or style of life, or of beliefs and ideals—we might say, ‘It was not I who did that, but an earlier self’. (Parfit, p305)^4^. Paul cites becoming a parent as a paradigmatic example of a transformative experience.^17^ In Jenny’s case there might be the additional, even more profoundly transformative, experience of bereavement. A news article describing a real case of a free-birth choice that ended badly, cites a woman whose baby died following planned free-birth. The mother described vividly her subsequent guilt, and her conclusion in retrospect: ‘I think I brainwashed myself with the internet’.^18^ Her description of a profound shift in perspective marries with the notion that the self who experiences the harm in such cases might be different, to an important degree, from the earlier self who made a harmful choice.^16^

On an Identity Relative Paternalistic account, the reasons to be paternalistic would be stronger for Advance resuscitation refusal and Hospital birth refusal, than for Vaccine refusal. Since we would not permit harm to other people in the third-party variations of those cases, we potentially should not permit John and Jenny to refuse treatment. Or, at the very least, we should be more inclined to overrule or disallow their decisions. However, that would not apply to James’ Vaccine refusal.

In contrast, a time-neutral paternalistic account would treat these cases as symmetrical. Since we would not allow third parties to harm other people in these ways and for these reasons, we should allow neither James nor Jenny nor John to harm themselves in the ways that they intend.

If we take a reductionist approach to identity, which version should we adopt, which is most plausible?

There is some reason to think that Parfit would have supported Identity relative Paternalism. The reductionist identity morality and paternalism claims as articulated by Parfit relate to the harm that individuals do to their ‘future selves’. (Parfit, p320–1)^4^ In support of these claims he provided examples where the prudential unity relations are weakened. For example a boy starting to smoke and causing great suffering fifty years later. (Parfit, p319)^4^ However, it is worth being clear whether and why the moral reasons not to harm oneself apply only to far future selves and not our more proximate selves.

Parfit gave two alternative ways of expanding the scope of moral theory to include harm to our future selves. The first would be impersonal. The moral reason not to self-harm is because it results in reduced overall well-being or greater suffering. From an agent-neutral consequentialist perspective, the loss of well-being due to self-harm is the same as the loss of well-being incurred when a third party is harmed.^17^ The second alternative is agent relative. We could expand our understanding of our special duties and obligations (to kin, to friends, to clients) to include a duty to the self.^18^

If we expand the scope of morality in these ways, that would provide a basis for the reductionist identity moral claim and the corresponding paternalism claim. Yet on either basis, that would potentially apply equally to the proximate future self and the distant future self. From an impartial consequentialist perspective it would be just as harmful for John to make an unwise advance decision that shortens his life if the harm accrues shortly after his advance directive or many years later.^19^ Likewise, it is not clear why our agent relative duty to self applies to our far future and not our near future self.

If the moral reasons not to harm do not change over time, or with weakening of psychological connections, that would appear to support Time-neutral Paternalism. But it would be worthwhile returning to why the Reductionist Identity account provides support for paternalism in the first place.

When we recognise that what matters to us (in terms of future selves) is a matter of degree, that may change what we have reason to care about in an egoistic way. We may then come to care less about harms that occur to ourselves in the far future. It would not be irrational to make imprudent decisions if the harms will occur at a time later when the psychological connections to our current self will be relatively weak. (Parfit, p313–4)^4^ The Reductionist Identity account weakens the prudential reasons that we have to avoid harms to future selves. But it does not, itself, generate a corresponding moral reason to intervene.

**Figure 1:**
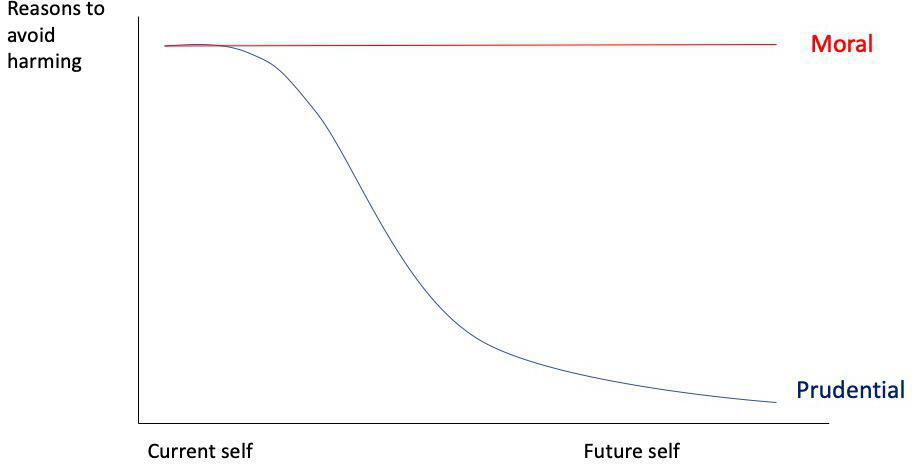
The strength of reasons to avoid harm and the time point of harm occurring. Prudential reasons are “reason(s) from the prudential point of view”.^28^ The moral reasons to avoid harm do not change over time, but the prudential reasons may diminish (where prudential unity relations are weaker). The individual does not necessarily regard their actions as harmful, and may regard it as in their self-interest to eg avoid a transfusion or give birth away from a hospital. However, those self-interested (prudential) reasons potentially attenuate in strength the further in the future the self that would be impacted by the decision.

Where does the case for paternalism come in then? As argued, the moral reasons not to harm, are not identity relative, they are time-neutral. What the Reductionist Identity account does is not to create a moral reason to avoid harm to a future self—rather it potentially unmasks those moral reasons. Figure 1 illustrates this.

As the figure indicates, for proximate self-harming, there are both prudential and moral reasons not to harm the near-future self. For far-future self-harming, the prudential reasons (currently) may be relatively weaker insofar as the psychological connections and continuity are diminished, while the moral reasons remain the same.

On this model, it seems that the reductionist identity moral claim should be time neutral. We ought not to do to our future selves (whether or not we are closely psychologically connected to those future selves) what it would be wrong to do to other people. But there is a further question about the Reductionist Identity Paternalism claim. That is because for proximate future selves there is a potential conflict between the prudential reasons not to harm (as perceived by the individual) and the moral reasons. We need to consider then the difficult question of how to balance prudential and moral considerations.

## Paternalism and the duality of practical reason

Sometimes we face choices between what we believe would be best for us, and what would be impartially best. For example, it may be that we could help someone else, but only at a significant personal cost. Or it may be that we could take a course of action that would be better for ourselves, but at the cost of failing to do what we morally (impartially) ought to do.

The moral philosopher Sidgwick regarded the potential conflict between these two different types of reasons as ‘the profoundest problem of ethics’. (Sidgwick, p386)^20^ The problem, as Sidgwick saw it, was that such reasons are not straightforwardly comparable. It is not clear how much sacrifice of personal well-being we are required to make for the sake of impartial beneficence. There is no external viewpoint that would allow us to answer such a question. (Parfit, p133–4)^21^

According to Sidgwick, if we have a choice between what would be impartially best and what would be best for ourselves, it would be rational to take either choice—this is what he referred to as the ‘dualism of practical reason’.^20^

The dualism might not be thought to apply to paternalism cases like the first-party treatment refusal cases cited above. In these cases, the prudential reasons to avoid harm and the moral reasons actually coincide. Indeed, the reason that we might be tempted to be paternalistic is because of concern for the individual’s well-being. So there is no conflict between incompatible reasons.

However, from the point of view of James and Jenny and John, the prudential reasons do diverge. They believe that the decisions they are making would be best for their current and future selves. They believe that they would be harmed by accepting the vaccine, transfusion or hospital birth. They appear to be mistaken, but from their perspective that is not the case. (Indeed, they are not likely to even recognise this conflict since they would regard the choices they are making as promoting the well-being of their future selves).

The question for paternalism is whether health professionals or societies are justified in overruling an individual’s personal judgement about what would be best for himself on the basis of a reliable concern that this would in fact cause the future person great harm. This does seem to potentially be a conflict between a type of prudential reason and a moral one. (A familiar way in medical ethics of characterising this conflict would be to see this as a clash between ‘autonomy’ and ‘beneficence’).

One response, drawing on the dualism of practical reason, would be to say that there is no way to weigh up or arbitrate between these two types of reason. Sidgwick, himself, could identify no way of balancing these reasons. In that case, faced with any degree of conflict between prudential and moral, autonomy and beneficence concerns, we may decide to give primacy to autonomy and reject paternalism. Indeed, the conventional response to treatment refusal cases takes exactly that path. No matter how great the well-being cost, or how irrational the reason, so long as the individual has the capacity to decide, they should be permitted to refuse treatment.

Yet, many philosophers who have followed Sidgwick have rejected his pessimistic conclusion that no answer could be found that would balance the two types of reason. Parfit’s own discussion of Sidgwick emphasised that he thought that Sidgwick’s account depended on the rational significance of personal identity.

Given the unity of each person’s life, we each have strong reasons, Sidgwick claims, to care about our own well-being in our life as a whole. And given the depth of the distinction between different people…one person’s loss of happiness cannot be compensated by gains to the happiness of others.[32, 133]

But this was, according to Parfit, to overstate the importance of personal identity. (Parfit, p136)^21^ On the reductionist identity account, our reasons to care about our future are based not on the fact that such a future is ‘ours’ - rather on the basis of the psychological relations between our current and future selves. Moreover, Parfit contended that we can have some similar partial reasons to care for the well-being of other individuals connected to us (friends and relations) and separate impartial reasons to care about everyone’s well-being.

Parfit’s own preferred way of addressing the dualism was through what he called the wide value-based objective view and the notion that such reasons *are* comparable, although only imprecisely.

Wide value-based objective (WVO) view If one of two possible acts would make things go impartially better, while the other act would make things go better from a partial perspective (for ourselves or someone close to us), we could have sufficient reason to act in either way. (Parfit, p137)^21^

Drawing on the WVO view, Parfit concluded that strong impartial considerations could sometimes outweigh weak prudential ones. We would have much stronger reasons to save many strangers from death or agony than to save ourselves from some minor harm. (Parfit, p137)^21^ For a practical example, on one plausible interpretation of this view, a passing stranger would have stronger reason to save a child drowning in a pond, than to save his expensive suit.^21^

Parfit alluded to the relevance of a Reductionist Identity view for the dualism and as support for the WVO view. However, it also seems that we could draw on this WVO view in thinking about paternalism. The WVO is about moral as well as rational permissibility. One problem with time-neutral paternalism is that it appears to give no weight to the prudential reasons for acting—it takes only an impartial perspective. In contrast, identity relative paternalism would defer to the individual’s judgement for harms that are proximate, but potentially give greater weight to impartial moral considerations as the psychological connections between current and future-self diminish. This would potentially be supported by the idea, on the WVO, that strong impartial reasons could outweigh weaker prudential reasons. That suggests that paternalism would be most justified in cases where the harm will be accrued by a future self relatively psychologically distant from the current person.

## Practical implications of identity relative paternalism

I have defended Identity relative paternalism. What would such a view mean for the harm principle and for refusal of medical treatment?

According to the view I have described, if we would not allow an individual to refuse treatment for a third party (eg, where they are a surrogate decision maker), we should potentially disallow refusal of treatment where the harm will be accrued by a future self. How strong a reason there is to act paternalistically will depend on the relative strength or weakness of prudential unity relations. The sharp boundary between harms to self and harms to third parties would be dissolved.

### Expiry of advance directives

Such a view could lead to questioning of advance directives written a very long time prior to their application, as occurred in advance treatment refusal. In fact, this already appears to be supported to some degree in practice.^22^ Clinicians are asked to consider whether there is reason to think that the individual might have changed their mind since completing their advance directive.^22^ Patients are encouraged to review their advance directives periodically (for example every 2 years).^23^ However, we could take that further. Advance decisions that are older than a certain period and have not been formally reviewed could lose their legally binding status.^23^ They could still be taken into account, but their status would change. They would then simply indicate the views of the patient at an earlier time point. They might be regarded in the same way as the views of next of kin or family members are for patients without capacity—relevant but not necessarily binding. Where it is overall in a patient’s best interests to treat contrary to a much earlier advance directive, that could be authorised—in just the same way that an authorised surrogate decision-maker can be overruled if making decisions contrary to a patient’s best interests.

It might be thought that this reductionist argument (in favour of potentially ignoring advance refusals of treatment with the passage of time) would apply a fortiori to ignoring advance refusals of treatment in cases of severe brain injury or dementia.^24^ That might mean that patients are unable to write binding advance directives that apply to their future self with dementia. However, while such cases involve significant psychological discontinuity, they are complicated by changes in the capacity of the individual. It certainly appears that the prudential unity relations are significantly weakened. However, it is also extremely difficult or impossible to know what the wishes or values or views of the later self would be. One approach would be to give priority to the wishes of the earlier self (because of their greater autonomy or moral status). Another approach would be to consider the views of the earlier self as akin to the views of a close family member. That might plausibly lead to a presumption in favour of following those wishes, but would also allow overriding a prior advance directive that would be clearly harmful to the later self (for example refusal of pain relief or palliative care, or a demand for burdensome treatment despite little/no prospect of benefit).

### Compelling treatment

What of cases like Home birth refusal, where harms will potentially occur in the near future, but we may have reason to think that psychological connections will be weakened. Such cases are more difficult. That is partly because of the challenge of prediction. Not all individuals change in their outlook and perspectives when they become parents, or when they are bereaved, or to the same extent. There may be considerable uncertainty about whether sufficient weakening of prudential unity will occur to warrant paternalistic intervention. There is an important question about whether we should assume prudential unity and give priority to the wishes of the current individual, or assume prudential disunity and prioritise preventing harm to the later self.

There is a further complication. Let us assume Jenny_T2_ is sufficiently different from her earlier self to warrant treating this as a ‘harm to others’ case. It does not follow that it would be justified to compel her to have medical treatment (eg, a caesarean section). That is because we would not necessarily inflict certain forms of treatment on individuals even to prevent harm to third parties. Consider the example in box 7.

Box 7 **Case: Liver donation refusal**

Abel has severe liver failure and is listed for transplantation. However, he has a relatively rare tissue type and he lives in a country where there are relatively few deceased donor livers. It seems likely that he will die waiting for a transplant. Abel’s brother Cain is the only closely matching family member who would be suitable for a living partial liver donation. This donation would have a high chance of helping Abel and relatively low risk to Cain. However, Cain declines to donate.

We would not compel Cain to donate part of his liver to Abel—even though it would (in the example) prevent severe harm to another. This applies to significant surgical intervention (donating part of a liver, or a kidney). It also arguably would apply to blood transfusion. Someone ought to donate blood to prevent serious harm to a third party. But we would not force them to do so.^25^ This suggests that we need to modify our principle.

Identity relative paternalistic intervention: Individuals should be prevented from doing to future selves (where there are weakened prudential unity relations between the current and future self) what it would be justified to prevent them from doing to others.

On the basis of the identity relative paternalistic intervention principle, it would not be justified to perform a caesarean section on Jenny against her wishes, since we would not justifiably perform major surgery on one person for the sake of another individual.^26^

However, some less intrusive steps might be permitted by this modified intervention principle. For example, some states mandate vaccination.^24^ That is typically justified on the basis of prevention of harm to third parties. Vaccine mandates are controversial. Yet, if they can be justified on that basis, it could also be possible to vaccinate paternalistically where the harm will accrue to a future self (with weakened prudential unity relations).^27^ That would not apply to James’ case, (since we might expect the greatest risk of COVID-19 is in the short term), but it could apply in other situations where the illness prevented is in the further future (for example with the human papilloma vaccine to prevent future cervical cancer). Here is another possibility: I have focused on patient refusal of treatment. But we might imagine another case where a patient requests treatment that would cause harm to a third party. It would be justified for doctors to refuse to perform surgery or to provide a treatment that would harm a third party. Correspondingly, they might also decline to provide a treatment that would harm a future self (even if there is not separate harm to a third party).

### Acutely life-threatening choices

In Vaccination refusal, James is potentially at risk of dying if he contracts severe COVID-19. Although it would plausibly be in James’ best interests to have a vaccination contrary to his expressed wishes, the future James harmed by intervention (ie, who would die) is psychologically close to the current James. James_t2_ is not akin to another person. Mill’s harm principle would apply to a case like this.

There is a potentially significant difference between acutely life-threatening harms and other harms. For example, one possibility is that James would not die even if he developed severe COVID-19. But he might survive with other serious complications (for example, he might have a cardiac arrest and develop hypoxic brain injury). If James were to survive, there could then be a future self who would be sufficiently psychologically distant from the current James, and who would have been harmed.^28^ This suggests a potential paradox: Identity-relative paternalism might be more permissive of acutely life-threatening choices than of choices that are not life threatening but would lead to survival long term in a harmed state.^29^ While this conclusion may be surprising, it is not without precedent. For similar reasons, concern about harm to the future child can provide a stronger reason to intervene with maternal choices that lead to survival of an impaired fetus than with maternal choices that might lead to death of the fetus.^25^ This could mean that although it permits paternalistic intervention, Identity relative paternalism would not support prohibition of assisted suicide, even where the doctor has reason to believe that the individual patient’s future life would be worth living. This paradox is related to a familiar conundrum in relation to reproduction—the so-called ‘Asymmetry’.^26^ ^30^ In this case, there are identity relative paternalistic reasons to intervene where there will be a future (psychologically more distant) individual who is harmed. However, those reasons do not apply in situations where (absent intervention) the future individual will not exist. One possible response to this would be to claim that in cases like that of James, (where his vaccine refusal is life-threatening) doctors/courts would be justified in compelling vaccination since this will lead to (or increase the chance of) existence for a future James_t10_ who will then be psychologically distant from James_t1_. However, if we do so, that is not to prevent harm to others. Future James_t10_ may benefit from our intervention, but he would not have been harmed had we allowed James_t1_ to refuse the vaccine, since this future James_t10_ would not have existed.

## Objections

### Concerns with paternalism

Some will reject identity relative paternalism on the basis of familiar objections to interventions in individuals’ lives. Decisions made on behalf of another person are potentially fallible and prone to abuse. Decisions about medical treatment involve values as well as scientific facts, and there can be reasonable disagreement about what would be best.

However, these sorts of concerns also apply to third party decisions made for incompetent patients. In that setting, they are not taken to mean that we must avoid decisions for patients who are unable to decide for themselves, rather that such decisions should be taken with great care.^31^ What is more, these concerns about fallibility and abuse also apply to the decisions that individuals make about their future selves. Individuals may fail to take into account the interests of their future selves, they may be mistaken about what those future selves would care about, or fail to give sufficient weight to those future interests. Disregard for our own future well-being could be regarded as a form of elder abuse or even a type of discrimination against a class of individuals who are unable to protect their own interests—in a similar way to unfair treatment of future generations. The epistemic and consequentialist arguments against paternalism do not succeed in establishing a sharp difference between future-self harm and other-harm.

### Rejection of reductionist identity

Others will reject the above arguments because they are sceptical of the reductionist claim that our future selves are relevantly like other people. For example, if someone believes in a Cartesian ego, or a soul, then it is clear that there is a binary answer to questions of identity and a bright line between the self and others. Still others may reject the Parfitian view because they regard some of its radical implications as a reductio ad absurdum.

Parfit himself admitted that his view was revisionary. He noted that it yielded some implications that were contrary to conventional views and potentially controversial. The notion that paternalism is more easily justified is one such implication. However, it is worthwhile also noting that the alternative view is also counterintuitive and unattractive. The three examples given in this paper of refusal of medical treatment might be uncommon or unusual, yet in some situations patients do make decisions that are profoundly unwise and risk great harm to their future selves. Health professionals in such circumstances often feel deeply conflicted—perhaps precisely because they recognise that such choices are morally wrong even if they are decisions (as things currently stand) that the individual patient has a legal right to make.

### Vagueness and uncertainty

I have suggested that there is a difference between decisions that harm a near-future self, and those that harm a far-future self. However, drawing this distinction might be problematic. As explicitly endorsed by the reductionist identity account, there is no clear boundary between near future and far future selves. Rather, there is a continuum characterised by greater connectedness at one end, and lesser connectedness at the other. Moreover, there is likely to be uncertainty about how much personal change an individual will undergo over time.^32^ How will we know when to intervene?

This type of concern is a perennial problem in practical ethics. Ethical considerations or reasons often exist in a spectrum, and boundaries are frequently vague. Predicting the impact of decisions on future individuals can be challenging and uncertain. However, in one way, this is a virtue, not a weakness of the reductionist paternalism account. Even on the conventional Millian account, there is a need to weigh up the degree of harm to others that would be caused, and the other countervailing reasons not to intervene. The reductionist account indicates another important consideration to be weighed.

### Non-identity

Finally, an additional complication is that certain patient choices may not merely cause a future self to have medical complications and reduced well-being. The choices may change in a fundamental way the nature of the future self who experiences them. For example, if future Jenny_t2_ (following a complicated home birth) has been ‘transformed’ by the experience of perinatal loss, there might be a question about whether she has been harmed in a counterfactual sense. After all, if the current Jenny_t1_ had given birth in hospital, that would have given rise to a different future self.

This raises the possibility that considerations of future selves might give rise to complicated new forms of Parfit’s non-identity problem.^4^ As Parfit famously noted, in special cases where our decisions that would affect which future individuals would be born, we sometimes cannot say that a specific future individual is worse off, since the alternative is that they would not exist.

However, decisions that affect our future selves are importantly different from those that affect which future individuals exist. On the reductionist account, our future selves may be more like other people than we conventionally think. Nevertheless, (absent exceptional circumstances) there are physical and psychological continuities between the current and future self. These mean that the current self can have an interest in the well-being of their future self. It also means that the future self can coherently claim that they have been counterfactually harmed—even where their life would have gone radically differently had their younger self made a different choice. It would be completely coherent for Jenny_t2_ to lament the choice of Jenny _t1_, though it would not make sense in a typical non-identity case for a child (who has a life worth living) to lament their parents’ choice to conceive them rather than a different (healthier) child.^33^

## Conclusions

In this paper, I have argued that if we adopt a reductionist account of personal identity, the bright line between harm to self and harm to others becomes blurred and the Millian harm principle fails to generate a clear prohibition against intervening to prevent future harm. I have described two different forms of paternalism potentially arising from a reductionist view of identity and suggested that in the face of conflicting prudential and moral reasons, a wide value-based objective view supports a form of Identity relative paternalism. I have defended a new identity relative paternalitic intervention principle. The point is not that identity relative paternalism necessarily or always justifies paternalistic interventions in such cases, rather that there is a stronger ethical case for doing so than is often recognised. Harm to self can be sufficient to warrant state intervention—where that harm is significant, and that future self is, to a relevant degree, like another person.

Pace Mill, power (including medical power) can be rightfully exercised over competent adults, against their will for their own benefit. The strong moral reasons to prevent harm to other people can also apply to our future selves.

## Data Availability

Data sharing not applicable as no datasets generated and/or analysed for this study.

## Footnotes

This might also apply if the evidence that vaccination prevents transmission weakens—as appears to be the case with the omicron variant of COVID-19.^29^

Others have observed some of the implications of Parfit’s account for applied ethics and paternalism. For example, Jeff McMahan, in The Ethics of Killing, notes that weakening of the prudential reasons to care for one’s far future weakens the arguments against paternalism. (McMahan, p288)^30^ Cyril Hedoin has explored some of these issues.^31 32^ He argues that the conventional idea of a trade-off between autonomy and beneficence in instances of soft paternalism is mistaken, because a reductionist identity account (or Parfit’s separate notions of Rational consent) diminish the significance of personal autonomy.

for example, as defined by Kuhse.^9^ If these arguments succeed, they will also apply to soft paternalism.

For example, Ben Davies has argued in favour of paternalism in cases of predictable changes in future evaluative outlook.^33^ Reductionist paternalism is potentially compatible with other arguments supporting paternalism. It may be, for example, that in some cases the arguments I present (in relation to personal identity and future selves) do not apply, but other reasons would support paternalistic intervention.

One complication (which I will not have space to explore) is that in some cases, refusals of treatment can also potentially cause harm to other people—for example if they lead to costly complications that then need to be treated in a publicly funded healthcare system.

NB Some philosophical debate has cast doubt on the distinction between harm to self vs harm to other people in Mill’s harm principle. Mill himself admitted that actions will often affect both the self and other people. (Mill, p145)^2^ Some philosophers have suggested that it is better to distinguish not between harm to self and harm to other people, but between self-regarding actions and non-self-regarding actions.^34^

Mill excluded from this principle those who are not ‘in the maturity of their faculties’. He contended that those who require care from others should be protected from harm from their own actions (as well as from harm from other people). Ibid p22-23.

‘With respect to his own feelings and circumstances, the most ordinary man or woman has means of knowledge immeasurably surpassing those that can be possessed by any one else’. (Mill, p137)^2^

The General Medical Council in the UK states emphatically that doctors ‘must respect a competent patient’s decision to refuse an investigation or treatment, even if you think their decision is wrong or irrational’. (GMC, p24)^11^

The legal status of these three decision makers is different, depending on the jurisdiction. In all three cases, however, even if they had legal authority the surrogates would be ethically and legally required to make decisions in the best interests of the patient.

Other philosophers who have defended forms of paternalism have also suggested that the strong asymmetry implied by the harm principle may not be justified, though for different reasons. Eg.^36^

According to Parfit, what matters morally is what he calls ‘Relation R’, and he regarded Relation R as being a function of ‘psychological connectedness and/or continuity with the right kind of cause’. (Parfit, p262)^4^ In Parfit’s view, this included memories, but also beliefs, desires, intentions and character traits. Reductionist paternalism as described in this paper is not dependent on a particular theory of Relation R.

Tim Campbell has argued that the interesting normative implications are a result of the combination of the Reductionist view and the view that what matters morally is psychological continuity. Campbell refers to this as Psychological Reductionis.^37^

McMahan distinguishes between personal identity (which is necessarily all or nothing), and the rational basis for egoistic concern, which he calls prudential unity (which may come in degrees). It is partly for this reason that McMahan and Parfit hold that identity is not what matters for egoistic concern. (McMahan, p39–43)^30^

Parfit’s claim might need to be modified, since it could be interpreted too broadly. I return to this in the section on practical implications.

Although I cite here Paul’s description of transformative experience, Identity Relative Paternalism is not dependent on a particular account or definition of such experiences. Experiences may transform individuals to greater or lesser extents, and correspondingly may weaken to a greater or lesser degree the psychological connections between earlier and later selves.

This will not always hold. In some cases, when a third party is harmed, both they and those around them may experience more distress than if the individual had harmed himself.

Mill recognised the importance of self-regarding virtues.(Mill, p136)^2^ However, he did not think that they could generate a duty as they were not enforceable.(Mill, *Ch5 para 14*)^19^ Joseph Kranak has argued that duties to the self should be understood as duties to future selves—drawing on the reductionist idea (which he refers to as a metaphysical fiction) that the self is not unified over time.^38^

In a different way, a later harm may be less harmful—since it will potentially result in shorter period of reduced or foregone well-being

Crisp notes that strictly speaking, Sidgwick did not regard a conflict between egoism and utilitarianism as yielding a sufficiency of reasons—rather to practical chaos. (Crisp, p230)^39^

Parfit did not (to my knowledge) discuss Peter Singer’s famous pond example, but it seems plausible to interpret his view in this way.

For example, the Code of practice relating to the UK Mental Capacity act is clear that ‘If the person’s current circumstances are significantly different from those when the decision was made, the advance decision may not be applicable’. MCA CoP 9.51^40^ (It is worth noting that rejecting an advance directive because someone appears to have changed their mind, is different from rejecting an advance directive because the self to whom it applies is psychologically distant from the self who wrote it. Both might be justified, but for different reasons.)

Some jurisdictions (eg, Oregon) allow patients to indicate an expiry date for Advance directives. However, I am not aware of any that currently apply an automatic expiry.

See for example Dresser.^6^

Even in an emergency situation, with a national shortage of blood donors, we would not usually compel people to donate blood.

In this example, intervention might prevent harm to two other individuals—Jenny’s future self, and her future child.^25^ I have hitherto set aside taking into account the interests of the future child. However, if that were included, it would still not necessarily justify intervention. For example, a single blood donation is often separated into different components (red blood cells/plasma/platelets) and can be used to help more than one person. However, we do not ordinarily think that it would be justified to compel someone to give blood even to prevent harm to more than one individual. Correspondingly, it would not be justified to compel a person to undergo significant surgery even to prevent harm to more than one other person.

One example of a vaccine where refusal will potentially cause harm to a future self (but not necessarily harm to third parties) is tetanus—since this is usually not transmissible to others, and herd immunity does not apply.

Future James might have weakened psychological connections with the current James either because the experience of serious illness and subsequent disability is transformative, or simply because he will live for long enough that a sufficiently distinct future self will emerge.

This would hold even if the impairment or illness were less harmful than dying.

There can be moral reasons not to bring into existence an individual who will have a life not worth living (it would be potentially morally wrong and harmful to do so), though there are not moral reasons to bring someone into existence who will have a life worth living (and it would not be morally wrong or harmful to fail to do this).

As noted earlier, such concerns are not sufficient reason to permit third party harm.

For example, will a future James retain his anti-vaccine beliefs, or will his mind have changed? How much will Jenny’s views, values, and traits change?

There is a further response to this objection. Even if it were the case that the non-identity problem applied to future selves, it does not follow that ‘harmful’ choices are thereby automatically unproblematic. Parfit defended what he called the ‘no-difference’ view in relation to non-identity cases. (Parfit, p369)^4^ On this view, the absence of a counterfactual harm should make no difference to our moral judgement about decisions that will predictably lead to reduced well-being in future individuals.

One complication of this case is that Jenny’s decision will affect both her and her child. For the sake of this paper, I wish to set that aside. My focus here is on whether the reasons *for Jenny’s sake* alone are sufficient (or indeed provide any reason) to justify compulsion.

There are a number of people who have been inspired by Rhinehart’s novel to make risky decisions on the basis of dice rolling.^35^
